# Willingness to Pay a Higher Price for Pork Obtained Using Animal-Friendly Raising Techniques: A Consumers’ Opinion Survey

**DOI:** 10.3390/foods12234201

**Published:** 2023-11-21

**Authors:** Carlo Giannetto, Vito Biondi, Annalisa Previti, Angelina De Pascale, Salvatore Monti, Angela Alibrandi, Agata Zirilli, Maurizio Lanfranchi, Michela Pugliese, Annamaria Passantino

**Affiliations:** 1 Department of Economics, University of Messina, Via dei Verdi, 75, 98122 Messina, Italy; giannettoc@unime.it (C.G.); adepascale@unime.it (A.D.P.); mlanfranchi@unime.it (M.L.); 2 Department of Veterinary Sciences, University of Messina, Via Umberto Palatucci, 98168 Messina, Italy; vito.biondi@unime.it (V.B.); annalisa.previti@yahoo.it (A.P.); salvatore.monti@unime.it (S.M.); annamaria.passantino@unime.it (A.P.); 3 Unit of Statistical and Mathematical Sciences, Department of Economics, University of Messina, 98122 Messina, Italy; angela.alibrandi@unime.it (A.A.); agata.zirilli@unime.it (A.Z.)

**Keywords:** animal welfare, consumers, willingness to pay, animal welfare, pork

## Abstract

In Italy, the consumption of pork meat is increasing, despite consumers’ attitudes being addressed toward a greater sensitivity about animal welfare and its link with safe food. Considering the relatively high animal welfare standards and the divergence in public interest in farm animal welfare and ethical issues, the objective of this study—in continuation of our previous paper relating to consumer behavior and preferences in welfare-friendly pork breeding—was to investigate habits of pork consumers regarding pig welfare, principally evaluating their willingness to pay (WTP) a higher price for pork obtained using raising techniques with an approach based on animal welfare. An ad hoc questionnaire-based survey was submitted to consumers (n = 404) in Messina province, Italy. Results suggest that 47% of consumers were willing to pay an additional price for pork from farms that apply specific animal welfare standards. Positive correlations were between WTP and farming techniques (*p* = 0.001), organic farming methods (*p* = 0.001), and farms in which animal welfare is taken care of and guaranteed (*p* < 0.001). These findings suggest that consumers intend to pay a higher price for pork, like other animal products obtained using animal-friendly raising techniques. The sensitivity to the animal welfare of a single human being may influence consumers’ attitudes toward pork consumption.

## 1. Introduction

Meat consumption trends vary significantly across the world. The European average per capita consumption of pork is the highest among meat commodities [1]; in 2020, the average European consumed around 33.79 kg of pork, followed by 25.40 kg of poultry, 13.54 kg of beef/buffalo meat, 1.65 kg of sheep and goat, and only a lower fraction (1.45 kg) of other meat types [1]. Based on the Food and Agriculture Organization of the United Nations, it is noted that in the last twenty years, the global annual per capita consumption of pork has decreased by 3.82%, reducing from 38.01% annual consumption per capita in 2000 to 34.19% in 2020 [1]. The reasons attributable to this decreasing tendency can essentially be explained by the change in diet style and by the increase in food intolerances or allergies [2,3]. For example, vegetable alternatives are preferred, or white meat or fish is preferred to red meat [4,5,6]. The European consumption of pork reflects the global one, reducing by 2.87% from 2000 to 2020 [1]; on the other hand, in Italy, pork represented, in 2000, 42.24% of the annual consumption of meat per capita, increasing to 46.84% in 2020 [1]. Paradoxically, while there is an increase in Italy in the consumption of pork, the trend for other types of production is decreasing [1].

The COVID-19 pandemic has also contributed to making consumers more aware of the links between health, ecosystems, supply chains, consumption patterns, and the environment [7,8]. Consumer habits in food can also be influenced by the credibility and reliability of information sources, including government agencies, healthcare professionals, scientists, or social media. The level of trust placed in these sources or entities can determine whether a consumer chooses to follow their advice [7]. However, as far as the per capita consumption of pork is concerned, a different phenomenon has occurred: as mentioned above, pork consumption has substantially increased, and in Italy, it stands at around 38 kg per year. The percentage of Italians that consume pork at least once a week is equal to 45% [9], and Italian farms are mainly intensive [8]. According to the latest ISTAT data (2022), the consumption of pork, both fresh cuts and cured meats, shows how the effects of the recent economic crisis that has hit Europe have influenced households’ food purchasing decisions [10,11]. In fact, today, food consumption is oriented toward cheaper products to compensate for the increases and additional costs coming from the third sector [12,13]. Costs related to the third sector are mainly related to the environment, cultural entertainment, health services, assistance to people with disabilities, and the management of essential services provided to citizens. According to the latest Italian Institute of Services for the Agro-food Markets (ISMEA) report, pork sales in volume grew by 4.6%, with a consequent increase in expenditure in value of 10.3% [14].

Despite this trend, awareness of the issues of environmental sustainability and food safety is growing more and more steadily among the Italian population [15,16]. It is not surprising that numerous consumers are expressing concerns about the environment but fail to consistently act upon them. While consumers’ attitudes toward environmental sustainability are generally positive, there exists a significant disparity between favorable attitudes and the actual purchase of sustainable food products. This discrepancy is commonly referred to as the attitude–behavior gap [17]. Nevertheless, as previously mentioned, consumer habits are changing and are moving toward greater sensitivity in health and ethics [17,18]. Indeed, an increasing number of European consumers prefer ethical production systems and appear unwilling to buy products that do not meet their animal welfare concerns [19]. In fact, differences in attitudes and willingness to pay for welfare-friendly products across socio-demographic characteristics were observed. The available literature indicates that women, younger individuals, pet owners, and those with higher education and income levels exhibit heightened levels of concern and a greater propensity to invest in welfare-friendly products [20,21]. Notably, the younger generation emerges as particularly relevant, as they display heightened concern and a greater willingness to pay for welfare-friendly products [17]. Given their influence, they are poised to become the primary catalysts driving the future food market. In addition, consumers consider Farm Animal Welfare (FAW) strictly linked to the food quality concept and use it as a possible indicator of other attributes associated with human health and safety [22]. Moreover, consumers view organic production systems as more welfare-friendly, with higher standards of FAW than conventional livestock systems, and also as better for human health due, e.g., to the low use of chemical treatments [19]. However, at a national level, the evidenced interest in FAW is not reflected in a good communication strategy on labels, and to date, it is still difficult to identify animal-friendly products on the shelves [23]. Indeed, it is evident that consumers face considerable challenges in accurately identifying animal-friendly products. This difficulty arises primarily due to the voluntary nature of labels based on higher animal welfare standards in the European market. Furthermore, the absence of a unified certification system that encompasses the entire EU territory leads to a proliferation of diverse labels. Consequently, producers effectively communicating their additional commitment to animal welfare to consumers can prove to be problematic [23]. The challenge of identifying animal-welfare-friendly products becomes even more pronounced when considering the stages following farming, such as transportation or pre-slaughter. These phases have a clear potential to alter the quality of pork [24], as demonstrated by the observed variation in meat quality parameters that are directly influenced by the stress encountered during pre-slaughter handling [25].

Ethical principles are aimed at the psycho-physical well-being of the animal and possess an environmental sustainability factor, such as greater attention to the impacts of intensive farming [26,27]. As well, within the most interesting issues related to FAW, some studies have investigated consumers’ acceptance of several farming practices, such as surgical or immunological castration in pigs [28,29,30,31].

Today, more and more consumers, at the time of purchase, place greater importance on guaranteeing FAW [18,19,20,21,22,23,24,25,26,27,28,29,30,31,32]. In fact, within the Italian agro-food system, paths are being created that are oriented towards sustainability, respect for the land, enhancement of culture and traditions, and attention to social issues [33,34]. This broad and profound metamorphosis, in contrast with the globalized food system, also responds to the need to meet the recommendations of Agenda 2030 and the indications contained in the Farm to Fork (F2F) Strategy. Indeed, the 2030 Agenda affirms the need to make changes to restore the balance between production and consumption. The F2F Strategy, together with the Biodiversity Strategy for 2030, is at the heart of the European Green Deal program [35,36,37]. With these strategies, the EU has developed a commitment to creating an agri-food sector that is increasingly sustainable from a social, environmental, and economic point of view.

These considerations show the need to create a responsible governance approach between the various sectors connected to agri-food production, agriculture, livestock, forestry, fishing, environmental and energy policies, and rural and forestry development [38,39]. A multidisciplinary and multistakeholder approach is needed that allows the implementation of ambitious and shared objectives aimed at reinforcing the existing links between the areas of agricultural and livestock production, transformation, and, consequently, consumption. In fact, in accordance with consumer guidelines, the ethical management of breeding is for the first priority for animal welfare.

In this paper, the authors continue their previous study published [40] in which consumers’ behavior and preferences in relation to welfare-friendly pork breeding were explored. It describes some results of a survey carried out by the Department of Veterinary Medicine and the Department of Economics of the University of Messina and is aimed at investigating the perceptions and knowledge of the Italian consumer concerning the concept of animal welfare in pig farms and respects the link between animal welfare and food quality.

Furthermore, an attempt is made to investigate and analyze consumer behavior and their potential willingness to pay (WTP) a higher price for pork obtained from farms that respect animal welfare.

## 2. Materials and Methods

### 2.1. Compliance with Ethical Standards

The study included human participants who responded to a questionnaire. Consent complying with the Regulation (EU) 2016/679 [41] regarding the processing of personal data and Italian legislation [42,43] and the Legislative Decree. no. 33 of 14.03.2013 was collected.

### 2.2. Sampling Design, Tools, and Data

In order to collect the data, an ad hoc questionnaire was administered (available in the Supplementary Materials). It consisted of three sections. The first section contained questions relating to the socio-demographic characteristics of respondents (age, gender, educational qualifications, employment status, and income range). The second section was related to variables measuring respondents’ sensitivity toward animal welfare and aimed to explore the respondents’ knowledge of this issue, such as the importance attributed by respondents to farming techniques respecting animal welfare, i.e., those that avoid mutilating practices or practice castration techniques other than surgical. A short paragraph containing information about pig welfare on the farm was given to consumers in the guidelines of the questionnaire.

The information given below presents high-welfare practices, meaning farming practices allowed by current legislation [44,45] focusing on the mutilating practices and castration techniques (Box 1) such as castration with general anesthesia, immunocastration, embryo sexing, and genetic selection.

Box 1 **Summary of identified high-welfare practices relating to mutilating practices and to the castration techniques in pig species.**

**Focus on mutilating procedures**
The intervention carried out for reasons other than therapeutic or diagnostic purposes or for the identification of the pigs in accordance with Council Directive 2008/120/EC and resulting in damage to or the loss of a sensitive part of the body or the alteration of bone structure shall be prohibited
**Exceptions according to Directive 2008/120/EC**


**Advantages**

**Disadvantages for**

**Docking of a part of the tail**
Prevents injuries to other animals; safety reasonsPain and stress**Uniform reduction of corner teeth of piglets** by grinding or clipping not later than the seventh day of the life of the piglets, leaving an intact smooth surface; boars’ tusks may be reduced in length where necessary Prevents injuries to other animals; safety reasonsPain and stress
**Nose-ringing**
Better practice when the animals are kept in outdoor husbandry systemsPain and stress
**Castration of male pigs (without anesthesia)**
Cheap and fast; prevents “boar taint”Extremely painful and stressful for pigs even several days after surgery
**Proposed alternative procedures to castration**

**Castration with local anesthesia**

**and analgesic**

**Castration with general anesthesia**

**Immunocastration**

**Embryo sexing**

**Genetic selection**
Less stress and pain for the piglet compared to the traditional technique, but requires veterinary staff, and piglets show stress at the inoculation of drugsNo stress for pigs; more expensive; requires veterinary medical staff; piglets at risk of hypothermia upon waking up; requires hospitalization of at least 5 hNo pain or stress; the effectiveness of the vaccination plan and the quality of the meat need to be checkedExpensive and specializedSelection of subjects with low androsterone production (decreased fertility and more expansive)Any of the procedures described above shall only be carried out by a veterinarian or a person trained as provided in Article 6 of the Directive and experienced in performing the applied techniques with appropriate means and under hygienic conditions. If castration or docking of tails is practiced after the seventh day of life, it shall only be performed under anesthetic and additional prolonged analgesia by a veterinarian.

To evaluate consumer WTP for pork produced under high-welfare practices, in the third section of the questionnaire, four intervals (as percentage increase) were listed as follows: 0–5%, 5–20%, 20–50%, and 50% more; respondents were asked to select the above intervals that they believed corresponded with their WTP. We considered WTP very weak if it fell within interval 1, weak if it fell within interval 2, moderate if it fell within interval 3, and strong if it fell within interval 4.

The sampling design was simple random sampling, which is probabilistic. It guarantees representativeness because it is based on the total random enrollment of statistical units. The reliability of the questionnaire was guaranteed through the administration of a pretest on a small sample of 42 statistical units, selected by a random procedure in different areas of Messina province (a town in the region of Sicily, Southern Italy), trying to preserve the representativeness of sex (20 males and 22 females) and age (mean 37.4 ± 7.8 years). To carry out the research sample and collect a large catchment area, ensuring the presence of different types of subjects, the questionnaire was administered near very busy places (supermarkets, main squares, universities, etc.) in a random way within several municipalities in Messina province. The survey took place between September and December 2022. The anonymous questionnaire was directly distributed using the face-to-face method. Completion of the surveys required approximately 10 min per consumer.

A total of 450 questionnaires were collected. Out of the participants, 35 were removed because they asked about the option of having their results excluded from the analysis immediately after the survey. Furthermore, although the recruitment was for pork-eaters, in the original dataset, there were 11 vegetarians and vegans; these were also removed. Both elimination procedures have been commonly used to improve data quality in surveys.

The final sample size consisted of 404 respondents (42.8% male and 57.2% female), with a mean age of 37.9 ± 12.3 years.

### 2.3. Statistical Analysis

The categorical variables were expressed as absolute frequencies and percentages, and the numerical parameters (age, importance attributed to farming techniques, importance attributed to organic farming methods) as mean and standard deviation.

To investigate possible statistically significant differences between two groups of subjects defined by the variable WTP more (Yes or No), the Mann–Whitney test was applied for numerical variables and the Chi-square test for categorical variables.

Univariate logistic regression models were estimated to individuate the factors that significantly influence the WTP a higher price for pork from farms where animal welfare is respected [46]. As it is known, logistic regression is a specific case of Generalized Linear Models (GLMs) and represents a methodologically adequate solution when there is a need to identify a set of potential predictive factors of a dichotomous outcome (response variable) that assumes only two modalities. A multivariable logistic model was also estimated to identify independent significant predictors of WTP. Age, gender, educational qualification, income range, number of family members, religious believers (yes or no), the importance attributed to farming techniques, the importance attributed to organic farming methods, and preference for farms oriented towards animal welfare (yes or no) were the examined covariates in both approaches.

The results of univariate and multivariate logistic regression models were reported as Odds Ratio (ORs), 95% Confidence Interval (95%C.I.), and *p*-value.

A *p*-value lower than 0.05 was considered statistically significant and reported in bold in all tables.

Statistical analysis was performed by using SPSS for Windows Package, version 22.0.

## 3. Results

Table 1 shows absolute frequencies and percentages for categorical variables and Table 2 shows the descriptive statistics (mean ± standard deviation) for numerical variables related to the whole sample.

As can be seen from the absolute frequencies and percentages of the categorical variables shown in Table 1, the sample is mainly composed of women (57.2%), with a diploma (62.4%), coming from an urban area (82.4%), mainly unmarried (70%), with several family members equal to three or four (64.1%), with an average income between EUR 10,000 and 29,999 (48%), with a religious belief (71%), who are very willing to spend more for the purchase of pork from farms that protect animal welfare (88.6%), and who prefer sustainable companies (86.1%).

As reported in Table 2, the average age of the respondents is equal to 37.9. Moreover, examining the scores given by the respondents (on a scale from 0 to 10) to “Importance attributed to farming techniques respecting animal welfare” and “Importance attributed to organic farming methods”, we observed a score higher than 7 for both factors, denoting that the importance attributed is quite high.

**Figure 1:**
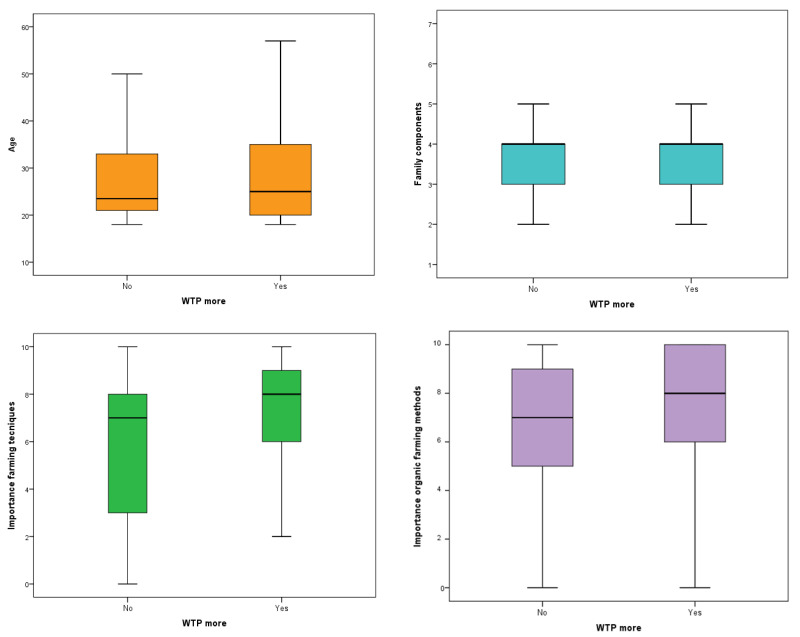
Boxplots of numerical variables according to WTP more (Yes or No).

**Figure 2:**
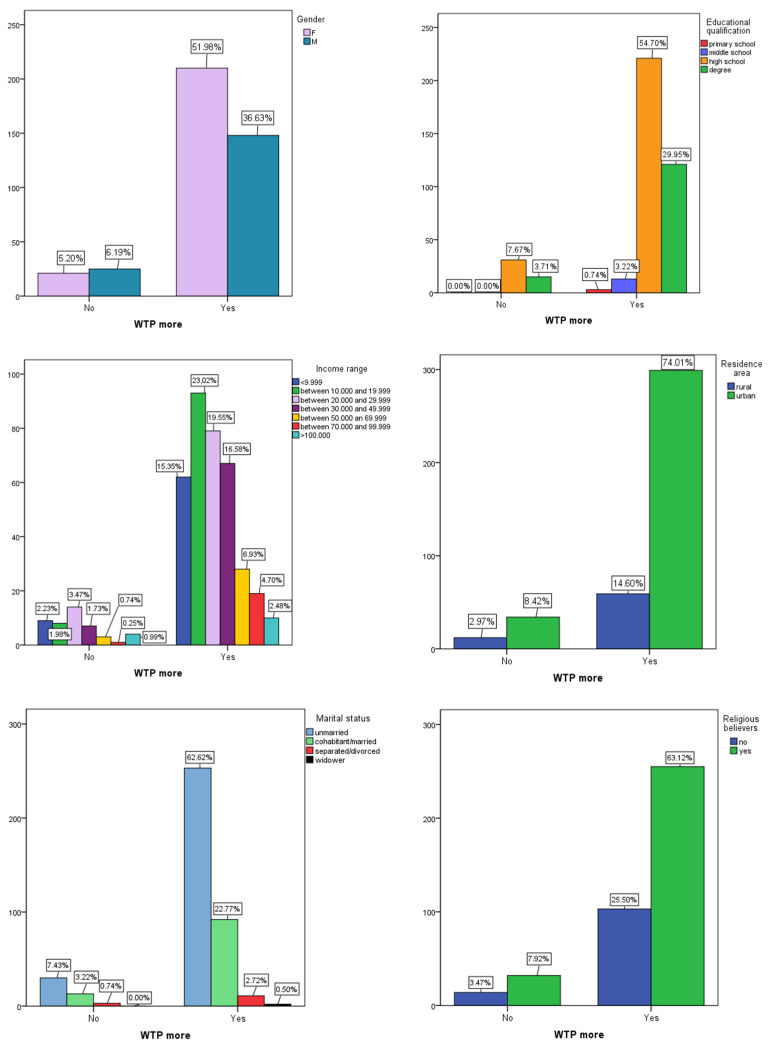
Bar charts of categorical variables according to WTP more (Yes or No).

Figure 1 and Figure 2 show box plots for numerical variables of interest (sex, income class, etc.) across Yes and No groups for WTP more and bar charts for categorical variables, respectively.

As shown in Figure 1, the Mann–Whitney test—applied to the numerical parameters—revealed a condition of similarity between those who are willing to pay more for animal welfare (WTP more Yes group) and those who are not (WTP more No group) in relation to the variables age (*p* = 0.874) and number of family members (*p* = 0.443). On the contrary, the test highlighted significant differences between the two groups in relation to the variables “Importance farming techniques” (*p* = 0.004) and “Importance organic farming methods” (*p* = 0.005) with significantly higher values in the Yes group.

Figure 2 demonstrates that no statistically significant difference was found between the two groups (WTP more Yes or No) regarding all categorical variables (*p* > 0.050).

Table 3 shows the results of univariate and multivariable logistic regression models for WTP more (Yes/No) for the purchase of pork from farms oriented towards animal welfare.

The results of the logistic regression model, both univariate and multivariate, allow us to state that there are significant predictors of WTP for the purchase of pork from farms oriented towards animal welfare since the *p*-values of some variables are statistically significant (*p* < 0.050).

More specifically, univariate models allow us to identify as significant predictors the importance attributed to farming techniques (OR = 1.187; *p* = 0.001), the importance attributed to organic farming methods (OR = 1.175; *p* = 0.001), and the preference for farms in which animal welfare is taken care of and guaranteed (OR = 2.883; *p* < 0.001). The Odds Ratio values greater than 1 for all three variables indicate that the respondents who assign great importance to these aspects are willing to pay more for the purchase of pork from farms where animal welfare is protected.

The socio-demographic results show that variables relating to age, gender, educational qualification, and income range do not have a significant effect on WTP (*p* > 0.050). In fact, the multivariate model confirms the significance of only two variables; the factors that significantly affect the WTP more are the importance attributed to breeding techniques (OR = 1.185; *p* = 0.001) and the preference for farms oriented towards animal welfare (OR = 2.642; *p* = 0.002).

However, the analysis carried out shows that 47% of consumers declare that they are willing to pay a premium price of more than 50% to buy pork that respects animal welfare techniques. The reasons that push the consumer to pay an additional price are mainly linked to respect for the animal (70.3%) and the quality of the product (19.8%); less frequently encountered reasons are the safety of the product (7.2%) and the non-consumption/purchase of meat (2.7%).

## 4. Discussion

These findings suggest that consumers intend to pay a higher price for pork, like other animal products, obtained using animal-friendly raising techniques, according to other authors [47,48,49]. For consumers from Western countries, price is not the only determining factor behind animal-based food purchases, as they are acquiring a growing interest in farming practices and related animal welfare standards [19,50].

Animal welfare is also perceived with differing importance by consumers according to the different production systems of the EU (such as organic or traditional production systems) that are characterized by complex interactions of many different components such as housing, feeding, breeding, and health management [49]. Understanding the disparities in beliefs and attitudes toward animal welfare among consumers is crucial. It is imperative to provide consumers with comprehensive information that enhances their knowledge about the practices necessary to ensure animal welfare throughout the various stages of production. By doing so, it is possible to emphasize the significance of humane treatment towards animals and to highlight its direct impact on food safety [49]. Moreover, gaining insights into the diverse types of consumers and their specific attitudes and beliefs regarding animal welfare can greatly assist in designing effective campaigns and targeted marketing strategies on this subject matter [49].

Certainly, the COVID-19 pandemic has raised consumer awareness on topics addressing animal, human, and environmental health. Consumers look for humane, healthy, and environmentally friendly food. Therefore, a multimodal approach combining the mandatory requirements, corresponding to a level of animal welfare whose recognition is undisputed among citizens, with a voluntary certification related to animal welfare using a One Health approach could be desirable to offer further guarantees to consumers and satisfy their demands.

Dransfield et al. [51] have highlighted that consumers appeared to be prepared to pay an average of 5% extra for pork from outdoor-raised pigs, with one-fifth of consumers declaring to be willing to pay 20% extra. If more and more pork products could be marketed at premium prices, with a strong emphasis on upholding FAW standards, it would create a remarkable market substitution [51]. Consumers, recognizing the increased value placed on ethical treatment and FAW, would be willing to pay a higher price and, in our opinion, this shift in consumers’ demand would incentivize producers to prioritize and invest in comprehensive FAW measures. Some studies have revealed a direct relationship between improved animal welfare and higher-safety animal products. Moreover, the WTP a premium price for animal-friendly products may highlight consumers’ altruistic tendencies, showing their humanitarian concern for animals [52].

Unlike our survey, Yang et al. [53] showed that socioeconomic characteristics such as education level, income level, gender, and age significantly affect consumers’ WTP. The food safety concerns of consumers and perceived consumer effectiveness also influence consumers’ WTP.

The consumer’s attitude towards other animal species such as broilers has already been demonstrated. Without support from consumers, adequate FAW standards cannot be improved. If consumers are willing to pay the premium price of high-welfare production, market segmentation should be applied. However, if consumers do not want to pay an extra cost, then government regulation is needed to improve the welfare of farm animals and to prevent costs and other issues caused by intensive animal farming [54].

The consumer has awareness and sensitivity towards the issue of animal welfare. The greater awareness of consumers regarding animal welfare on farms, in pig farms, in fact represents a great potential to attract demand in the livestock market [55,56,57,58].

People’s perceptions can differ depending on what they think in their different roles as citizens and as consumers [59]. Consumers respond to economic incentives with individualistic and materialistic concerns by maximizing their utility and thus rationally choosing products. A citizen’s point of view can be based on other values more related to altruistic concerns [60]. The active participation of citizens in shaping public opinion, driving legislation, and influencing political decisions by expressing their concerns for FAW is a significant phenomenon. Through activities such as voting, writing letters to politicians and media, and engaging in associations, citizens contribute to the process of public opinion formation. This collective effort can potentially lead to changes in attitudes, behaviors, and opinions, thereby serving as a driving force for enhancing the ethical status of farm animals within society [19]. Consumers, on the other hand, hold a unique influence in the marketplace, as they possess the ability to modify their purchasing behavior or boycott products from systems that do not align with their preferences [19]. It is needed to specify, in line with Alonso et al. (2020), that the terms “concern” and “attitude” are sometimes used interchangeably, but they hold distinct meanings. In fact, “attitude” refers to a psychological inclination expressed through the evaluation of a specific entity with varying degrees of favor or disfavor; “concern” pertains to the evaluation or attitude towards a particular issue [19].

The gap between the consumer’s role and the citizen’s role remains a crucial issue. If consumers recognize that animal welfare products are healthier, they will be willing to pay premium prices for high-welfare products and thus adopting high-welfare practices will not result in farmers losing a competitive advantage due to increased production costs [61].

Although some studies on this topic have shown that the perception of consumers regarding animal welfare does vary significantly according to socio-demographic variables [53,54,55,56,57,58,59,60,61,62], our results contradict this trend.

Instead, the availability to pay more derives from factors linked to the sensitivity of the single individual and, therefore, to the personal importance that they attribute to animal welfare. For this typical respondent, it is essential that the animal does not undergo unnecessary suffering before its death and that it is raised with techniques that protect and promote animal welfare.

Animal welfare in breeding should therefore start from the review of consumer attitudes, from the moment of purchase and during consumption [19,63,64,65].

Indeed, the survey carried out shows that products from farms that respect the needs of pigs inspire consumer confidence at the time of purchase. For this reason, consumers are willing to increase their WTP and are willing to face a higher economic sacrifice than those products in which there is no clear link between breeding and animal welfare. The consumer’s WTP for an improvement in animal welfare is a topic widely discussed in the literature [20,66,67,68].

Although this propensity of the consumer provides useful information about what their attitude could be, it does not give indications about what their real behavior could be, which should be analyzed with appropriate investigation techniques. The study therefore relaunches a much debated topic in the literature, namely that the improvement of pig welfare should start with the reduction in intensive farming [69,70,71,72]. This consideration underlines the developments of the growing interest in human–animal relationships [73,74,75,76]. This link is receiving much attention in various scientific areas such as economics, veterinary science, biology, anthropology, psychology, geography, and other cultural studies [26,77,78,79].

This study also revealed that the majority of consumers, in line with what is stated in the literature, believe that there is an association between animal welfare and food quality [80,81,82,83].

This contribution also shows the need to inform the consumer about the farming practices adopted in Italy to protect animal welfare, as it is noted that there is a high information asymmetry. The synergy between institutions, scientific research, and operators in the sector (veterinarians, breeders, etc.), who, in various capacities, work for greater protection of animal welfare, is essential to encourage the adoption of informed food choices.

The current study has limitations due to a recruitment bias that could occur in that participants who have an existing interest in the topic may be more predisposed to take part in the study. Nevertheless, the survey reflected the views of a large sample representative of data demographics, thus providing rigor to the findings.

## 5. Conclusions

In conclusion, it would be interesting to apply a certification system based on the principles of One Health, like what the US already does by utilizing the so-called Health Certified^TM^ (OHC) for chicken and turkey products [78] (https://onehealthcertified.org/). Thereby, consumers would be guaranteed that pork is produced under a transparent program of best responsible care practices that the producers must follow to promote optimal health outcomes for animals, people, and the environment.

## Figures and Tables

**Table 1:** Absolute frequencies and percentages for categorical variables.

GENDER	
M	173 (42.8%)
F	231 (57.2%)
**EDUCATIONAL QUALIFICATION**	
Primary school	3 (0.7%)
Middle school	13 (3.2%)
High school	252 (62.4%)
Degree	136 (33.7%)
**RESIDENCE AREA**	
Rural	71 (17.6%)
Urban	333 (82.4%)
**MARITAL STATUS**	
Unmarried	283 (70.0%)
Cohabitant/married	105 (26.0%)
Separated/divorced	14 (3.5%)
Widower	2 (0.5%)
**FAMILY COMPONENTS**	
1	31 (7.7%)
2	45 (11.1%)
3	108 (26.7%)
4	151 (37.4%)
>4	69 (17.1%)
**INCOME RANGE**	
<EUR 9.999	71 (17.6%)
EUR 10.000–EUR 19.999	101 (25.0%)
EUR 20.000–EUR 29.999	93 (23.0%)
EUR 30.000–EUR 49.999	74 (18.3%)
EUR 50.000–EUR 69.999	31 (7.7%)
EUR 70.000–EUR 99.999	20 (5.0%)
>EUR 100.000	14 (3.5%)
**RELIGIOUS BELIEVERS**	
Yes	287 (71.0%)
No	117 (29.0%)
**WTP MORE**	
Yes	358 (88.6%)
No	46 (11.4%)
**PREFERABILITY OF FARMS**	
Yes	348 (86.1%)
No	56 (13.9%)

**Table 2:** Descriptive statistics (mean ± standard deviation) of numerical variables.

VARIABLES	M ± SD
Age	37.9 ± 12.3
The importance attributed to farming techniques respecting animal welfare	7.1 ± 2.8
The importance attributed to organic farming methods	7.4 ±2.7

**Table 3:** Univariate and multivariable logistic regression models for WTP: key findings.

COVARIATES	UNIVARIATE			MULTIVARIABLE		
	OR	I.C. 95%	*p*-Value	OR	I.C.95%	*p*-Value
Age	0.998	0.974–1.022	0.853	0.999	0.971–1.027	0.925
Gender (M vs. F)	0.592	0.319–1.097	0.096	0.710	0.357–1.414	0.330
Educational qualification	0.876	0.505–1.522	0.639	0.763	0.422–1.379	0.371
Income range	0.952	0.785–1.155	0.618	0.932	0.764–1.138	0.492
Residence area (urban/rural)	1.789	0.875–3.656	0.111	1.791	0.923–3.457	0.116
Family components	0.951	0.739–1.226	0.700	0.964	0.734–1.268	0.795
Religious believers (Yes/No)	1.083	0.555–2.113	0.815	1.095	0.526–2.281	0.808
Importance of farming techniques	1.187	1.077–1.309	**0.001**	1.185	1.072–1.311	**0.001**
Importance of organic farming methods	1.175	1.064–1.298	**0.001**	1.055	0.911–1.222	0.474
Preference for animal-welfare-friendly farms	2.883	1.652–5.030	**<0.001**	2.642	1.424–4.900	**0.002**

## Data Availability

The data presented in this study are available on request from the corresponding author.

## Supplementary Materials

The questionnaire can be downloaded at: https://www.mdpi.com/article/10.3390/foods12234201/s1.
