# Supplementary material for: Willingness to Pay a Higher Price for Pork Obtained Using Animal-Friendly Raising Techniques: A Consumers’ Opinion Survey
Source: Foods. 2023 Nov 21;12(23):4201. doi: 10.3390/foods12234201 (PMC10706721; doi:10.3390/foods12234201)
Supplement: Supplementary file 1 [file foods-12-04201-s001.zip › foods-2699551-supplementary.pdf]

Dear Participant,

This questionnaire is part of a scientific research project conducted by the Department of Veterinary Sciences in collaboration with the Department of Economics at the University of Messina. Our objective is to gauge public awareness of animal welfare, with specific emphasis on the mutilating practices carried out on pig farms.

Your participation in this study is completely anonymous and voluntary. Before proceeding with the questionnaire, please take a moment to read the introduction provided below.

Your data will only be accessed by authorized researchers and will be employed exclusively for statistical purposes, ensuring your anonymity and privacy. This complies with Articles 13 and 79 of Legislative Decree No. 196, dated June 30, 2003, known as the "Code on the Protection of Personal Data" (Privacy Code).

We greatly appreciate your valuable time and cooperation.

In Italy, two main types of pork are produced:

1. Lean or light pork, often referred to as butcher's pork, is typically slaughtered at around 110 kg.
2. Heavy charcuterie pig, usually slaughtered at approximately 140 kg and aged between 9 to 12 months.

In both cases, reaching sexual maturity may impact the sensory qualities of the meat due to what is commonly known as "boar taint" or "boar odor." This issue can often become noticeable when cooking or consuming these meats. Consequently, castration is often considered necessary and can be carried out through various methods:

- Traditional surgical castration (without anesthesia): This involves an incision of the scrotum and vaginal tunic to expose the testicles, which are then removed by tearing, severing, or twisting. This method is widely practiced due to its cost-effectiveness, speed, and lack of specialized personnel requirements. However, it is exceptionally painful and stressful for pigs, even several days post-surgery.
- Surgical castration with general anesthesia: This approach involves performing the surgical procedure after administering anesthesia. While it does not cause stress to the pigs, it is more costly and necessitates the presence of veterinary medical personnel. Piglets may risk hypothermia upon awakening and require at least 5 hours of hospitalization.
- Surgical castration with local anesthesia and analgesia: This method involves intratesticular anesthesia, often preceded or followed by the administration of slow-release analgesics. It causes less stress and pain for the piglets compared to the traditional technique but still requires the presence of veterinary medical personnel, and piglets may experience stress during drug administration.
- Immunocastration: This technique entails using a vaccine to delay the onset of sexual maturity, eliminating the need for surgical castration. The advantages include the absence of pain and stress. However, it necessitates testing the effectiveness of the vaccine plan and its potential impact on meat quality.
- Embryo sexing: This method involves selecting exclusively female embryos, eliminating the need for castration. It does not raise animal welfare concerns but comes with high costs and demands highly specialized veterinary personnel.
- Genetic selection of individuals with low androsterone production: This approach involves selecting pigs with reduced ability to synthesize scatol and androsterone, responsible for boar odor. While it can potentially reduce boar odor, it is associated with higher management costs and may affect androgen hormone production and the fertility of males.

Furthermore, various other mutilating procedures are carried out on farms to address management issues that could be mitigated by providing improved comfort to the animals, such as implementing quality environmental enrichment. However, these changes would likely result in increased costs for farmers, which could translate into higher meat prices.

Mutilations allowed under current regulations include:

- Tail severing
- Polishing or truncation of incisors in piglets, or when necessary, tusks in boars
- Application of a nose ring.

SECTION A - Socio-Demographic Information

1. Gender?
   - Male
   - Female
   - Other
2. Age? ……………………………………
3. What is your educational background?
   - No formal education
   - Elementary school degree
   - Middle school diploma
   - High school diploma
   - College degree (or higher)
   - Student in .... (specify year of study)
4. Nationality?
   - Italian
   - Other (please specify) …………………………..
5. City and Province of residence?
   - Urban area
   - Rural area
6. Marital status?
   - Single
   - Married
   - Cohabitating
   - Separated or Divorced
   - Widowed

7. Current employment status?

    - Unemployed
    - Homemaker
    - Employee
    - Self-Employed
    - Retired
    - Student

8. Which income bracket (after taxes) does your family fall into? (Your response is optional, but please note that the questionnaire is anonymous)

    - Under 9,999 euros
    - Between 10,000 and 19,999 euros
    - Between 20,000 and 29,999 euros
    - Between 30,000 and 49,999 euros
    - Between 50,000 and 69,999 euros
    - Between 70,000 and 99,999 euros
    - Over 100,000 euros

9. How many people are in your household (including yourself)? ________

10. Are you a believer?

    - Yes. Specify your religion ……
    - No

11. Which of the following categories do you belong to?

    - Consumer
    - Breeder or distributor (or professional in the meat industry)
    - Veterinary medicine student. Specify the current year …
    - Other (please specify): …………….

SECTION B - Consumption of Pork Meat

12. How important is pork meat consumption to you?

    - 1 Not important
    - 2
    - 3
    - 4
    - 5 Very important

13. How often do you consume fresh pork meat (not processed) per week?

 Never

 1-2 times

 3-4 times

 5-7 times

14. How often do you consume processed pork meat products (e.g., cold cuts) per week?

 Never

 1-2 times

 3-4 times

 5-7 times

15. Please indicate below the reasons why you prefer pork meat over other types of meat:

 Organoleptic qualities

 Price

 Tradition

 Easy preparation

 Other (please specify): ………………

16. Please rate the importance of the following factors in your pork meat purchase, assigning a score from 0 to 10 (0 = least important, 10 = most important):

	Score (0-10)
Product Price	
Specific leading brands in the industry (e.g.,Cremonini, Brianza, Sant'Angelo, etc.)	
Information on organoleptic characteristics	
Information on breeding techniques	
Exclusive Italian origin of pigs (100% Italian)	
Specific production region (location of the breeding) (e.g., Sicily, Calabria, etc.)	
PDO (Protected Designation of Origin) or PGI (Protected Geographical Indication) label	
Organic breeding method	

17. Do you consider yourself sensitive to the welfare of livestock animals?

 Yes

 No

18. Have you had work experience in the field of livestock farming?

 Yes

 No

19. What are your sources of information regarding pig farming?

 Institutional sources (universities, schools, specialized courses, etc.)

 Product labels

 Specialized magazines

 Television

 Internet

20. Before today, were you aware of the issue of boar taint?

 Yes

 No

21. Do you know what mutilations are allowed under current regulations?

 Yes

 No

22. Considering the information provided by us, do you believe castration of piglets is justified?

 Yes

 No

23. Considering the information provided by us, do you believe the use of mutilating practices is justified?

 Yes

 No

24. Considering the information provided by us, which mutilating practice do you support?

	YES	NO
Tail docking		
Smoothing or trimming incisors in piglets or, if necessary, tusks in boars		
Nose ring application		
Castration		

25. Considering the information provided by us, which method of castration do you support?

	YES	NO
Traditional surgical castration		
Surgical castration with general anesthesia		
Surgical castration with local anesthesia and analgesia		
Immunocastration		
Embryo sexing		
No castration		

26. Considering that mutilating practices (tail docking, incisor trimming, and nose ring application) can be avoided if pigs are raised with high welfare standards, even though this may impact the cost of the final product, would you be willing to spend more on the purchase of pork from animals raised with high welfare standards?

 Yes

 No

27. If NO, why not? (the questionnaire continues at question no. 31)

 It would be too expensive

 I don't think the quality is higher

 I don't believe it has a significant impact on my health or the animals' health

28. How much more would you be willing to pay for pork from pigs raised using non-surgical castration methods without anesthesia?

 0-5% more

 5-20% more

 20-50% more

 50% more

29. How much more would you be willing to pay for pork from pigs raised using on-farm methods that do not involve mutilations to enhance animal welfare?

 0-5% more

 5-20% more

 20-50% more

 50% more

30. For what reason would you be willing to pay more for the purchase of pork from pigs castrated using non-surgical methods without anesthesia?

 For product quality

 For product safety

 For animal welfare considerations

 Other (please specify)…………………….

31. For what reason would you be willing to pay more for the purchase of pork from pigs raised with on-farm methods that do not involve mutilations to enhance animal welfare?

 For product quality

 For product safety

 For animal welfare considerations

 Other (please specify)…………………….

32. How do you evaluate a pig farm that respects animal welfare at high standards compared to a farm that routinely practices mutilations?

 Worse

 Equal

 Better

33. Do you believe that pig farms that respect animal welfare are adequately advertised and supported?

 Yes

 No

 Indifferent
